# Supplementary material for: Vitamin D Modified DSS-Induced Colitis in Mice via STING Signaling Pathway
Source: Biology (Basel). 2025 Jun 18;14(6):715. doi: 10.3390/biology14060715 (PMC12190092; doi:10.3390/biology14060715)
Supplement: Supplementary file 1 [file biology-14-00715-s001.zip › Supplementary Materials S1: 16s-report of gut microbiota/Groups/betadiv/pca_analysis/PCA12_boxplot.pdf]

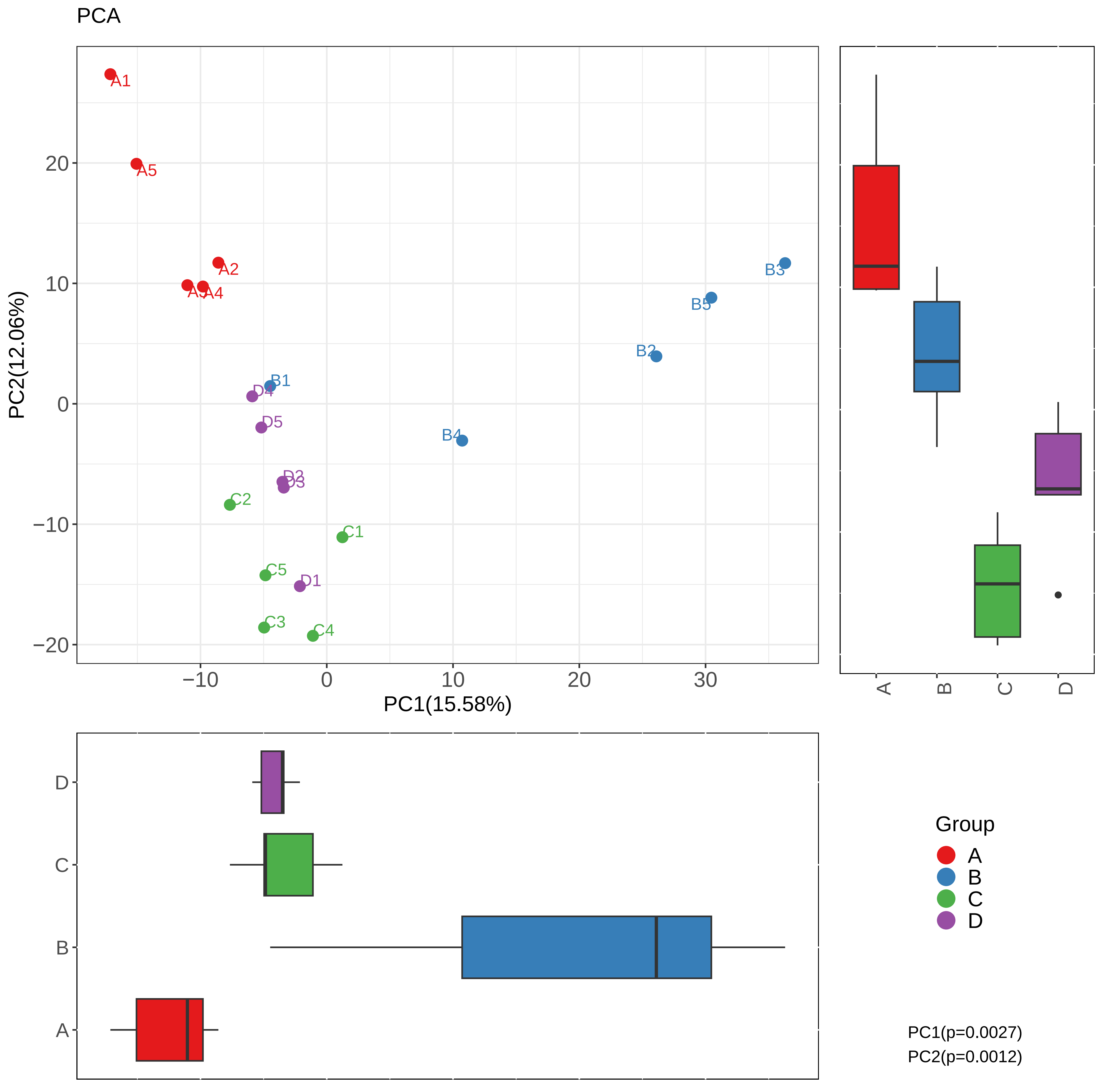

PCA
A1
A5
A2
A3
A4
B3
B5
B2
B1
B4
D4
D5
D2
D3
D1
C2
C1
C5
C3
C4
20
10
0
−10
−20
PC2(12.06%)
−10
0
10
20
30
PC1(15.58%)
A
B
C
D
Group
A
B
C
D
PC1(p=0.0027)
PC2(p=0.0012)
